# SIRT3 mediates hippocampal synaptic adaptations to intermittent fasting and ameliorates deficits in APP mutant mice

**DOI:** 10.1038/s41467-019-09897-1

**Published:** 2019-04-23

**Authors:** Yong Liu, Aiwu Cheng, Yu-Jiao Li, Ying Yang, Yuki Kishimoto, Shi Zhang, Yue Wang, Ruiqian Wan, Sophia M. Raefsky, Daoyuan Lu, Takashi Saito, Takaomi Saido, Jian Zhu, Long-Jun Wu, Mark P. Mattson

**Affiliations:** 1 Laboratory of Neurosciences, National Institute on Aging Intramural Research Program, Baltimore, MD 21224 USA; 2 Department of Neurology, Mayo Clinic, Rochester, MN 55905 USA; 3 Department of Pharmacology, School of Pharmacy, Fourth Military Medical University, Xi’an, 710032l China; 4 Laboratory for Proteolytic Neuroscience, RIKEN Brain Science Institute, Wako-shi, Saitama Japan; 5 Lieber Institute for Brain Development, Baltimore, MD 21205 USA; 6 Department of Neuroscience, Johns Hopkins University School of Medicine, Baltimore, MD 21205 USA

**Keywords:** Alzheimer's disease, Synaptic plasticity

## Abstract

Intermittent food deprivation (fasting, IF) improves mood and cognition and protects neurons against excitotoxic degeneration in animal models of epilepsy and Alzheimer’s disease (AD). The mechanisms by which neuronal networks adapt to IF and how such adaptations impact neuropathological processes are unknown. We show that hippocampal neuronal networks adapt to IF by enhancing GABAergic tone, which is associated with reduced anxiety-like behaviors and improved hippocampus-dependent memory. These neuronal network and behavioral adaptations require the mitochondrial protein deacetylase SIRT3 as they are abolished in SIRT3-deficient mice and wild type mice in which SIRT3 is selectively depleted from hippocampal neurons. In the *App*^*NL-G-F*^ mouse model of AD, IF reduces neuronal network hyperexcitability and ameliorates deficits in hippocampal synaptic plasticity in a SIRT3-dependent manner. These findings demonstrate a role for a mitochondrial protein deacetylase in hippocampal neurons in behavioral and GABAergic synaptic adaptations to IF.

## Abstract (web summary)

Intermittent fasting has been shown to have beneficial effects on hippocampal function in rodents, but the underlying mechanism is not fully understood. Here the authors show that the mitochondrial protein SIRT3 contributes to the beneficial cognitive and synaptic effects of intermittent fasting in mice.

## Introduction

Studies of laboratory rodents have shown that sustained intermittent food deprivation (IF, fasting) improves numerous health indicators, and counteracts disease processes in models of diabetes, vascular disease, cancers, and Alzheimer’s disease (AD)^1,2^. Randomized controlled trials of IF in overweight human subjects demonstrated improvements in multiple health indicators that suggest a potential for IF to reduce the risk of cardiovascular disease, diabetes, cancers, and inflammatory and neurodegenerative disorders^3–5^. Moreover, IF favorably impacts the disease processes in human patients with asthma^6^ and arthritis^7^. However, many individuals experience anxiety, irritability and difficulty concentrating on tasks on the fasting days when they first attempt IF^6,8^.

Animals have evolved complex metabolic and behavioral adaptations that enable them to maintain high levels of cognitive and physical performance in environments where food is relatively scarce and meals infrequent^9,10^. Humans who fast for extended time periods of a week or more experience heightened stress during the first few days, and then experience calmness and mental clarity during the remainder of the fast^11–13^. Subjects in controlled trials of IF (alternate day fasting or twice weekly fasting) report complete resolution of the initial side effects (hunger, agitation, irritability and headache) within 2–4 weeks of IF diet initiation^3,6,14^. The neuronal network-based mechanisms that presumably underlie the behavioral adaptations to IF eating patterns are unknown, but likely involve the hippocampus and amygdala, two brain structures that play major roles in responses to a wide range of environmental challenges/stressors^15–19^. Hippocampal neurons in rats or mice maintained on IF are relatively resistant to damage in experimental models of epilepsy^20,21^ suggesting that adaptation to IF may enhance constraint of neuronal network hyperexcitability. However, the underlying mechanisms are unknown.

Emerging findings suggest that mitochondria play important roles in adaptive responses of hippocampal neuronal networks to intermittent bioenergetic challenges, such as those occurring during exercise, fasting and performance of cognitively demanding tasks^10,22,23^. It was recently reported that the mitochondrial protein deacetylase sirtuin 3 (SIRT3) is upregulated in hippocampal neurons in response to exercise and protects mitochondria and neurons against metabolic and excitotoxic stress^24^. One of the mitochondrial proteins that is deacetylated by SIRT3 and may contribute to its neuroprotective actions is the antioxidant enzyme superoxide dismutase 2 (SOD2)^24^. In the present study, we provide evidence that neuronal networks in the hippocampus of mice adapt to IF by up-regulating GABAergic inhibitory neurotransmission via a mechanism requiring SIRT3 and involving a reduction in mitochondrial oxidative stress. These mitochondria-mediated molecular and cellular responses to IF reduce levels of anxiety-like behaviors and sustain cognitive performance. We further show that IF reduces hippocampal neuron hyperexcitability and ameliorates cognitive deficits in the *App*^*NL-G-F*^ mouse model of AD^25^ suggesting a potential therapeutic application of IF in AD.

## Results

### Behavioral adaptation to IF involves enhancement of GABAergic tone

All experiments were performed in male mice. Groups of wild type (WT), *App*^*NL-G-F*^ knockin and/or SIRT3 KO mice were assigned randomly to either intermittent fasting (IF) for either 1, 4 or 12 months and were then used for behavioral, electrophysiological and brain tissue analyses. Where indicated, mice were subjected to an acute 24-h period of food deprivation (FD) immediately before behavioral testing.

**Fig. 1:**
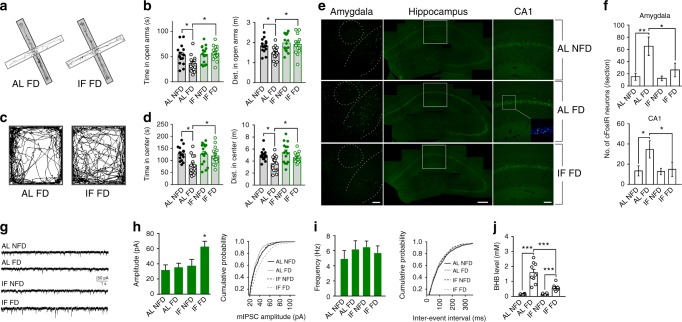
Behavioral and neuronal network adaptations to IF involve enhanced GABAergic activity. **a**–**d** WT mice were maintained on either an ad libitum diet (AL) or a on IF for 1 month. Mice in each diet group were then randomly assigned to behavioral testing in the elevated plus maze and open field after either a 24 h period of food deprivation (FD) or with no food deprivation (NFD). The images at the left show tracings of the walking paths of mice in the AL FD and IF FD groups in the elevated plus maze (**a**, **b**) and open field (**c**, **d**). The numbers of mice in each group were: AL NFD, 13; AL FD, 15; IF NFD, 12; IF FD, 15. *P < 0.05. **e** Examples of images of c-Fos immunoreactivity in neurons in the amygdala, hippocampus and CA1 of mice in the indicated groups. The dashed lines in the images of the amygdala demarcate the boundaries of the central nucleus (upper circle) and the basolateral amygdala (lower curved lines). The white frames in the images of the hippocampus demarcate the location of the images of region CA1. **f** Numbers of c-Fos immunoreactive (cFosIR) neurons in the amygdala and hippocampal CA1 in mice in the four indicated groups (5 mice/group). *P < 0.05, **P < 0.01. **g** Whole-cell recordings of miniature postsynaptic currents (mIPSCs) in CA1 neurons in hippocampal slices from mice in the four indicated groups. **h** Amplitudes of mIPSCs (left) and a plot of cumulative event probability as a function of mIPSC amplitude (right) in CA1 neurons of mice in the four different treatment groups (data are from 15 neurons in slices from 5 mice/group). *P < 0.05 compared to the values for each of the other three groups. **i** mIPSC frequency (left) and a plot of cumulative event probability as a function of mIPSC inter-event interval (right) in CA1 neurons of mice in the four different treatment groups (5 mice/group). **j** Serum β-hydroxybutyrate (BHB) concentrations in mice in the indicated groups. ***P < 0.001. All error bars are the SEM. All statistical comparisons used ANOVA and Newman–Keuls post hoc tests. Source data are provided in supplemental Source Data file

To determine the effects of IF on stress-related behavioral responses to food FD, mice were assigned randomly to either an ad libitum (AL) control diet or an alternate day IF diet and were maintained on the diets for 1 month (see Methods). Mice on the control diet that either were not or were food deprived for 24 h, and mice on the IF diet that were not or were food deprived for 24 h were then tested in the elevated plus maze (EPM) and the open field. After 24 h of FD, the mice that had been on the AL control diet spent less time and traveled less distance in the open arms of the EPM compared to non-fasted control mice (Fig. 1a, b). In contrast, the amount of time and distance traveled in the open arms for mice that had been on IF for 1 month and were then deprived of food for 24 h was not different than non-fasted mice. Similarly, AL control mice deprived of food spent less time and traveled less distance in the center zone of the open field compared to fed mice and to food-deprived mice in the IF group (Fig. 1c and d). The anxiogenic effect of acute FD in mice previously fed ad libitum was completely abolished in mice administered the GABA receptor agonist diazepam 1 h before the behavior tests (Supplementary Fig. 1a). We also found that the elevation of the serum concentration of the stress hormone corticosterone in response to restraint stress was significantly attenuated in mice adapted to IF compared to mice fed AL (Supplementary Fig. 1b).

Excitatory synaptic activity involves membrane depolarization and calcium influx through glutamate receptor channels and voltage-dependent calcium channels. Calcium then activates kinases that induce the expression of the immediate early gene *c-Fos*^26^. To determine whether FD affects the relative activation states of neurons in the amygdala and hippocampus, mice in the AL control diet group were either left in the fed state or deprived of food for 24 h, and brain sections were immunostained with a c-Fos antibody. We focused on the hippocampus and amygdala because of their well-established roles in behavioral responses to stressful situations^15–19^. Numbers of c-Fos immunoreactive (cFosIR) neurons were significantly elevated in the amygdala and hippocampus (CA1, CA3 and dentate granule neurons) of mice in the FD group compared to fed control mice (Fig. 1e, f and Supplementary Fig. 1c). In contrast, numbers of cFosIR neurons in the amygdala and hippocampus were not affected significantly by FD in mice that had been maintained on IF for 1 month (Fig. 1e, f and Supplementary Fig. 1c). As a positive control, we administered the GABA receptor antagonist picrotoxin to mice on the AL diet and found that it induced a dose-dependent increase in numbers of cFosIR hippocampal neurons, with 1.5 mg/kg eliciting a significant but submaximal response (Supplementary Fig. 1d). To mimic the excitatory increase caused by FD, we therefore treated mice with 1.5 mg/kg picrotoxin. Picrotoxin induced an anxiety-like behavioral response in mice on the control diet, but not in mice that had been on the IF diet for 1 month (Supplementary Fig. 1e). In an additional experiment, we found that intermittent low dose picrotoxin treatment (1.5 mg/kg, every other day for 1 month) abolished the anxiety-inducing effect of acute FD (Supplementary Fig. 1f). These results show that acute FD increases excitatory synaptic activity and associated anxiety-like behavior, which is abolished in mice adapted to IF or intermittent PTX administration.

GABA receptor agonists reduce anxiety and inhibits seizure activity^27^. To determine whether IF affects GABAergic synaptic activity, we performed electrophysiological patch clamp recordings from hippocampal CA1 pyramidal neurons in brain slices from mice that had been maintained on AL control or IF diets for 1 month. Recordings were performed under conditions that enabled isolation of miniature inhibitory (GABAergic) postsynaptic currents (mIPSCs) (see Methods). The amplitude of mIPSCs was unchanged in response to FD in mice in the AL control diet group (Fig. 1g, h). In contrast, the amplitude of mIPSCs was significantly increased in response to FD in mice that had been adapted to IF for 1 month (Fig. 1g, h). The frequency of mIPSCs was not affected by FD in mice in either diet group (Fig. 1i). Similar to mice in the IF group, hippocampal neurons of mice that had been administered low dose picrotoxin intermittently every other day for 1 month exhibited an increased amplitude (but not frequency) of mIPSCs in response to FD compared to mice administered vehicle intermittently (Supplementary Fig. 1g). These results suggest that mice adapted to IF exhibit reduced anxiety and attenuated stress responses that are mediated by enhancement of GABAergic neurotransmission.

Fasting results in a metabolic switch from utilization of glucose derived from liver glycogen stores to ketones derived from fatty acids^1^. To confirm that this metabolic switch was indeed occurring during the 24-h period of FD, we measured levels of the ketone β-hydroxybutyrate (BHB) in serum samples collected at the end of the 24-h fasting period in the AL-FD and IF-FD groups, and at the same time of day in the fed state in the AL-NFD and IF-NFD groups. As expected, BHB concentrations were increased greatly in the AL-FD and IF-FD mice compared to mice in the other two groups (Fig. 1j). Interestingly, the BHB concentration was elevated to a higher level in mice in the AL-FD group compared to mice in the IF-FD group. The reason that BHB levels are elevated less by fasting in mice adapted to IF compared to those not adapted to IF, may be that mice adapted to IF may have greater stores of glycogen as suggested by a recent study^28^.

### SIRT3 mediates behavioral and neuronal network adaptations to IF

**Fig. 2:**
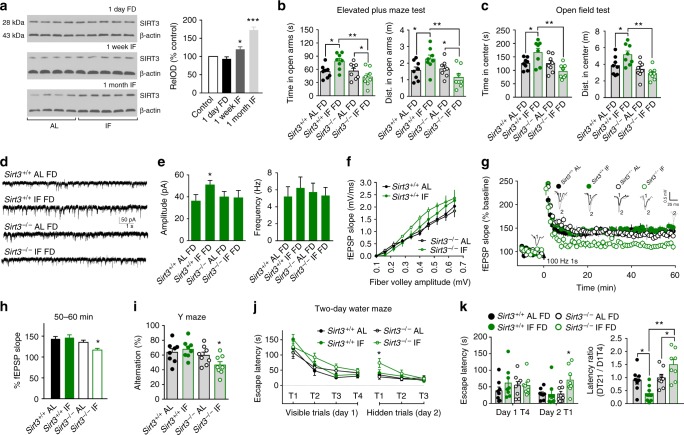
SIRT3 is required for behavioral and synaptic adaptations to IF. **a** Immunoblots show relative levels of SIRT3 protein in hippocampus of mice that had were either fed AL or maintained on alternate day food deprivation (IF) for 1 week or 1 month. The upper blot is samples from mice killed on the second day after diet initiation (at the end of the first 24 h FD period for mice in the IF group). *P < 0.05, ***P < 0.001 compared to the AL NFD and 24 h FD groups (11 mice/group). **b, c** Time spent (left) and distance traveled (right) in the open arms of the elevated maze (**b**) and in the center zone of the open field (**c**) for mice that had either been fed AL or IF for 1 month (12 mice/group). All tests were performed in mice that had been deprived of food for 24 h (FD). *P < 0.05. **d** Whole-cell recordings of mIPSCs in CA1 neurons in hippocampal slices from mice in the four indicated groups. **e** mIPSC amplitudes (left) and frequencies (right) of mIPSCs (15 neurons in slices from 5 mice/group). *P < 0.05. **f**–**h** fEPSP recordings at CA1 synapses in hippocampal slices from *Sirt3*^*+/+*^ and *Sirt3*^*–/–*^ mice that had been maintained for 4 months on either AL or IF diets. **f** shows basal synaptic transmission with the amplitude of the fiber volley plotted against fEPSP slopes (input/output curves). **g** shows the results of LTP analysis. Panel h shows quantification of LTP magnitude. *P < 0.01 compared to the values for each of other three groups (10 slices from 5 mice/group). **i** Results of Y-maze testing. **j**, **k** Results of water maze testing. Group difference, F (3, 27) = 3.762, *P* = 0.022. **k** shows data for escape latencies on testing day 1 trial 4, and testing day 2 trial 1 (left), and latency ratios (escape latency on day 2 trial 1 divided by the escape latency on day 1 trial 4) (right). 8 mice per group, *P < 0.05, **P < 0.01. All error bars are the SEM. All statistical comparisons used ANOVA and Newman–Keuls post hoc tests. Source data are provided in supplemental Source Data file

Mitochondria play roles in synaptic plasticity^29^, and it was recently reported that the mitochondrial protein deacetylase SIRT3 is up-regulated by running wheel exercise in the hippocampus of mice and mediates adaptive responses of neurons to exercise and metabolic and excitatory challenges^24^. To understand whether SIRT3 is also involved in the adaptive responses of neuronal network activity to IF, we performed immunoblot analysis of SIRT3 protein levels in hippocampal tissues and found that 24 h of FD in AL control mice had no effect on SIRT3 levels, and that mice maintained on IF for 1 month exhibited a highly significant elevation of SIRT3 levels (Fig. 2a). Hippocampal SIRT3 levels were also elevated significantly, albeit moderately, in mice that had been on the IF diet for 1 week. In contrast to the anxiolytic effect of IF in wild type mice, results of open field and elevated plus maze tests showed that the anxiety response to FD was not ameliorated by IF in SIRT3-deficient (*Sirt3*^*–/–*^) mice (Fig. 2b, c). In addition, whereas numbers of cFosIR neurons in the amygdala and hippocampus of mice following acute food deprivation were reduced in wild type mice adapted to the IF diet compared to those in the AL group, IF had no effect on numbers of cFos immunoreactive neurons in *Sirt3*^*–/–*^ mice (Supplementary Fig. 2a, b). Indeed, numbers of cFosIR neurons were significantly greater in *Sirt3*^*–/–*^ mice on the IF diet compared to *Sirt3*^*–/–*^ mice in the AL group. Recordings of mIPSCs in hippocampal CA1 neurons revealed that, in contrast to wild type mice, IF did not result in an increase of the mIPSC amplitude in *Sirt3*^*–/–*^ mice (Fig. 2d, e). Thus, SIRT3 is required for the adaptive enhancement of GABAergic synaptic transmission that occurs during adaptation to IF.

Rats or mice maintained on IF are able to sustain cognitive function and synaptic plasticity during aging, in contrast to animals fed *ad libitum*^30–32^. Because hippocampal neuronal circuits play critical roles in learning and memory, we determined the impact of SIRT3 deficiency on long-term potentiation (LTP) at hippocampal CA1 synapses. Field excitatory postsynaptic potentials (fEPSPs) were recorded in stratum radiatum of the CA1 region of the hippocampus in brain slices from *Sirt3*^*+/+*^ and *Sirt3*^*–/–*^ mice that had been maintained for 4 months on either AL or IF diets. Basal synaptic transmission and short-term potentiation, measured as the fEPSP amplitude during the first 5 min after high frequency stimulation, were not affected by genotype or diet (Fig. 2f, g and Supplementary Fig. 2c). LTP, measured as the fEPSP amplitude 50–60 min after high frequency stimulation, was not affected by IF in *Sirt3*^*+/+*^ mice, but was significantly reduced in *Sirt3*^*–/–*^ mice in the IF group compared to *Sirt3*^*–/–*^ mice in the AL group (Fig. 2g, h).

We next evaluated hippocampus-dependent spatial learning and memory in *Sirt3*^*+/+*^ and *Sirt3*^*–/–*^ mice that had been maintained for 4 months on either AL or IF diets using a Y maze test and a previously validated two-day water maze protocol^33^. Mice in all four groups gained weight during the 4-week period, but *Sirt3*^*–/–*^ mice on the IF diet gained less weight and had a lower food intake compared to mice in the other three groups (Supplementary Fig. 2d, e). In the Y maze test, *Sirt3*^*+/+*^ mice on either diet and *Sirt3*^*–/–*^ mice on the AL diet exhibited a typical alternation percentage (60–70%), whereas *Sirt3*^*–/–*^ on IF exhibited significantly reduced alternations suggesting a deficit in this test (Fig. 2i). In the water maze test, mice in all four groups readily found the visible platform on test day 1 (Fig. 2j, k). The results of the 3-trial hidden platform test on day 2 revealed that *Sirt3*^*+/+*^ mice in the IF group navigated to the platform location as readily as *Sirt3*^*+/+*^ mice in the AL group. However, *Sirt3*^*–/–*^ mice in the IF group took significantly longer to navigate to the platform location on hidden platform trial 1 compared to wild type mice and compared to *Sirt3*^*–/–*^ mice in the AL group (Fig. 2j, k). As an additional indicator of memory retention, we calculated the ratio of the goal latency on the first trial of day 2 to that of the last trial of day 1 (T1/V4) for each mouse. The results showed that the T1/V4 ratio was significantly lower in mice in the *Sirt3*^*+/+*^ IF group compared to mice in the *Sirt3*^*+/+*^ AL group, suggesting that IF enhanced memory retention in wild type mice (Fig. 2k, right panel). In contrast, the T1/V4 ratio was significantly greater in *Sirt3*^*–/–*^ mice on the IF diet compared to either *Sirt3*^*–/–*^ or *Sirt3*^*+/+*^ mice on the AL diet, suggesting a detrimental effect of IF on cognition in mice lacking SIRT3 (Fig. 2k, right panel).

To determine whether SIRT3 in hippocampal neurons is required for behavioral responses to IF, we used RNA interference technology to knock down (KD) SIRT3 in hippocampal pyramidal neurons of wild type (WT) mice. We injected AAV9-CAG-Sirt3-siRNA-GFP or AAV9-CAG-Control-siRNA-GFP into the dorsal and ventral hippocampus bilaterally in 2-month old WT male mice (Supplementary Fig. 2f). Previous reports showed that AAV9 selectively infects neurons in adult mouse brain, and we verified that the GFP was expressed in neurons. One month after AAV injection, we tested the mice in the elevated plus maze and open field and found that KD of SIRT3 in hippocampal neurons prevents behavioral adaptations to IF. The IF mice that received AAV9-Sirt3-siRNA-GFP showed greater anxiety-like behaviors after acute food deprivation compared to the IF mice that received AAV9-Control-siRNA-GFP (Supplementary Fig. 2g, h). These new data suggest that SIRT3 in hippocampal neurons is required to ameliorate anxiety-like responses to acute food deprivation in mice adapted to IF.

### IF ameliorates cognitive and synaptic deficits inAD mice

Human AD patients exhibit dysregulation of neuronal network excitability and increased incidence of seizures^34^, and the accumulation of Aβ increases the vulnerability of neurons to hyperexcitability and excitotoxicity in experimental models of AD^35,36^. In light of our evidence that IF enhances GABAergic tone, we therefore designed a study to determine whether IF would counteract neuronal network hyperexcitability and modify deficits in learning and memory and synaptic plasticity in an mouse model (Supplementary Fig. 3a) in which human APP with three different familial AD mutations is knocked into the endogenous mouse APP locus, thereby precluding non-specific effects resulting from random genome insertion and gene overexpression^25^. The *App*^*NL-G-F*^ mice develop several features of AD including deficits in spatial learning and memory associated with Aβ plaque deposition^37^. However, they do not exhibit neurofibrillary tangle-like pathology. As expected, *App*^*NL-G-F*^ mice in the present study developed progressive accumulation of Aβ plaques in the hippocampus and cerebral cortex (Supplementary Fig. 3b), and exhibited impaired performance in the Y maze when tested at 1 year of age (Supplementary Fig. 3c) compared to wild type mice. We randomly assigned 1 year-old wild type and *App*^*NL-G-F*^ mice to either AL or IF diets; for these experiments we employed a 2 days/week food deprivation IF regimen that is somewhat less stressful compared to alternate day fasting. We found that after two months of 2 days/week food deprivation IF, the old WT and *App*^*NL-G-F*^ mice showed similar adaptive responses to food deprivation in elevations of BHB levels and anxiety-like behaviors (Supplementary Fig. 3d–f).

**Fig. 3:**
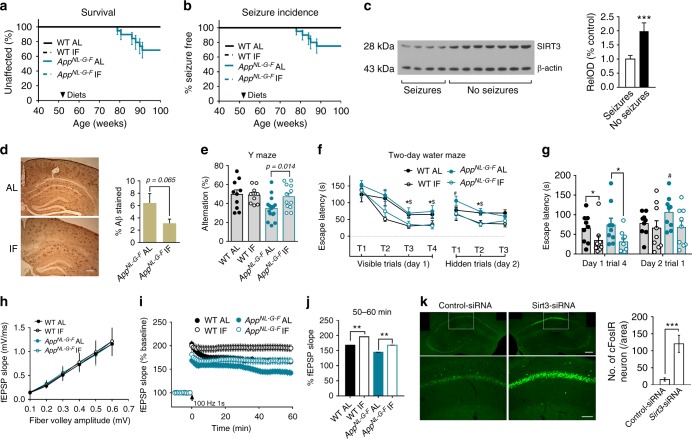
IF prevents seizures and ameliorates cognitive and synaptic deficits in *App*^*NL-G-F*^mice. **a** Survival curves for WT and *App*^*NL-G-F*^ mice maintained on either AL or IF (fasting 2 days/week) diets beginning at 52 weeks of age (WT AL, 10 mice; WT IF, 12 mice; AL *App*^*NL-G-F*^, 19 mice; AD IF, 11 mice. No WT mice or *App*^*NL-G-F*^ mice on the IF diet died, whereas 6 mice *App*^*NL-G-F*^ mice on the AL diet died. **b** Age of onset of first seizure-like behavior for mice in the four groups. **c** Immunoblot analysis of SIRT3 protein levels in hippocampus of *App*^*NL-G-F*^ mice in the AL diet group that either did or did not exhibit seizures. Densitometric analysis of SIRT3 band intensity (normalized to actin band; 4 mice with observed seizures, 7 mice without observed seizures). ***P < 0.001. **d** Images of Aβ immunoreactivity in hippocampus of *App*^*NL-G-F*^ mice that had been on either AL or IF diets for 8 months (scale bar, 100 µm). The graph shows Aβ load values (15 sections from 5 mice/group). **e** Results of Y-maze testing. Group difference, F (3, 32) = 5.538, *P* = 0.0035. *P < 0.05, WT-AL compared to values for WT-IF; ^$^*P* < 0.05, *App*^*NL-G-F*^ -AL compared to value for *App*^*NL-G-F*^ -IF; ^#^*P* < 0.05, *App*^*NL-G-F*^ -AL compared to each of the other three groups. **f**, **g** Escape latencies on testing day 1 trial 4, and testing day 2 trial 1. *P < 0.05 compared to the WT IF group; ^#^*P* < 0.05, *App*^*NL-G-F*^-AL compared to the other three groups (8–11 mice/group). **h**–**j** Field EPSP (fEPSP) recordings at CA1 synapses. Panel h shows basal synaptic transmission. **i** shows the results of LTP analysis. **j** shows quantification of LTP magnitude (10 slices from 5 mice/group). **P < 0.01. **k** c-Fos immunoreactivity in hippocampal CA1 neurons of an *App*^*NL-G-F*^ IF mouse one month after administration into the dorsal hippocampus of control shRNA lentivirus in the left hippocampus (left panels) and a lentivirus with an shRNA targeting Sirt3 RNA in the right hippocampus (right panels). (Scale bar, upper 250 µm, lower 100 µm). ***P < 0.001. All error bars are the SEM. Student’s *t* test was used for analyses of data in **c**, **d** and **k**. ANOVA and Newman–Keuls post-hoc tests were used for analyses of data in **e**, **f**, **g**, **i** and **j**. Source data for all graphs in this figure are provided in supplemental Source Data file

Within 9 months of diet initiation, 6 of the 19 *App*^*NL-G-F*^ mice in the AL group had died whereas none of the *App*^*NL-G-F*^ mice in the IF group had died (Fig. 3a). No WT mice in either diet group died during this study. One *App*^*NL-G-F*^ mouse in the AL group that died exhibited severe ocular inflammation, and the other five exhibited seizure-like behaviors (Fig. 3b). The seizure behaviors was associated with dramatically increased c-fos immunoreactivity in neurons in the cerebral cortex and hippocampus (Supplementary Figure 3g). SIRT3 levels in the hippocampus of *App*^*NL-G-F*^ mice without seizures were significantly higher compared to *App*^*NL-G-F*^ mice with seizures (Fig. 3c). There were no significant effects of genotype or diet on body weights and motor function (rotarod test) measured 8 months after diet initiation (Supplementary Figure 3h, i).

There was a non-significant trend (*P* < 0.065) towards lower amounts Aβ accumulation in *App*^*NL-G-F*^ mice on the IF diet compared to *App*^*NL-G-F*^ mice on the AL diet (Fig. 3d). One month prior to euthanizing mice for analyses of hippocampal synaptic plasticity and Aβ load, we tested their spatial learning and memory in the Y maze and water maze. *App*^*NL-G-F*^ mice in the AL group exhibited a significant impairment in the Y maze test compared to WT mice in the AL group (Fig. 3e). In contrast, *App*^*NL-G-F*^ mice in the IF group performed significantly better than *App*^*NL-G-F*^ mice in the AL group (Fig. 3e). Compared to WT and *App*^*NL-G-F*^ mice fed AL, those that had been maintained on IF exhibited significantly lower goal latency times in the water maze on training day 1 indicating that IF enhances spatial learning (Fig. 3f). On trial 1 on testing day 2, *App*^*NL-G-F*^ mice in the AL group exhibited a longer latency to reach the hidden platform compared with each of the other three groups (Fig. 3f, g). These findings show that eight months of IF ameliorates cognitive dysfunction in *App*^*NL-G-F*^ mice.

To determine whether the amelioration of hippocampus-dependent spatial learning and memory deficits in *App*^*NL-G-F*^ mice by IF was associated with corresponding improvements in synaptic plasticity, we evaluated basal synaptic transmission and LTP at CA1 synapses in hippocampal slices from WT and *App*^*NL-G-F*^ mice that had been maintained on AL or IF diets for 9 months. There were no significant differences in basal synaptic transmission among the four groups of mice as indicated by identical input-output curves (Fig. 3h). LTP was significantly reduced in slices from *App*^*NL-G-F*^ mice on the AL diet compared to WT mice on the AL diet (Fig. 3i, j). Compared to the corresponding genotype of mice in the AL groups, mice in the WT IF and *App*^*NL-G-F*^ IF groups exhibited a significant increase in LTP (Fig. 3i, j). To determine whether SIRT3 is required to constrain hyperexcitability in *App*^*NL-G-F*^ mice we used a lentiviral vector expressing a *Sirt3* mRNA-specific shRNA to knockdown SIRT3 expression in hippocampal CA1 neurons of *App*^*NL-G-F*^ mice. One month later we euthanized the mice and immunostained brain sections with the c-Fos antibody. Compared to sections from the same mice in other sides of hippocampal CA1 infected with a virus expressing a non-specific control shRNA, neurons infected with the *Sirt3* shRNA virus exhibited a marked increase in c-Fos immunoreactivity (Fig. 3k).

We next used lentivirus to knock down SIRT3 in hippocampal pyramidal neurons of *App*^*NL-G-F*^ mice that had been adapted to IF for 8 months. We then performed LTP analysis by electrophysiology and spine density quantification by Golgi staining in CA1 pyramidal neurons. We found that, one month after the onset of SIRT3 KD, the spine density of hippocampal CA1 pyramidal neurons was significantly less than the spine density in CA1 neurons of *App*^*NL-G-F*^ IF mice receiving the control lentivirus (Supplementary Figure 3j, k). We found that LTP at CA1 synapses was significantly less in CA1 neurons of *App*^*NL-G-F*^ IF mice receiving the SIRT3 KD lentivirus compared to those receiving the control lentivirus (Supplementary Figure 3l).

### SIRT3 is critical for GABAergic synaptic homeostasis

**Fig. 4:**
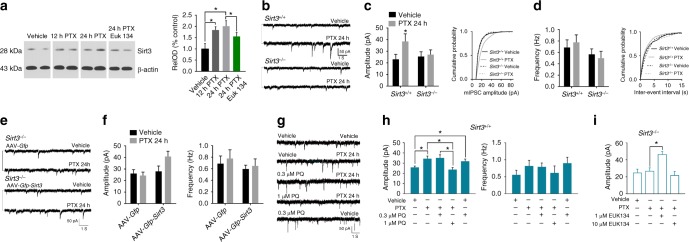
SIRT3 is required for GABAergic synaptic scaling. **a** Immunoblots (left) showing relative levels of SIRT3 protein in cultured WT hippocampal neurons exposed to the indicated treatments for 24 h; 100 μM picrotoxin (PTX) for 12 h; 100 μM PTX for 24 h; 10 μM EUK-134 plus 100 μM PTX for 24 h. (*P < 0.05). **b** Whole-cell recordings of mIPSCs in cultured hippocampal neurons from *Sirt3*^*+/+*^ and *Sirt3*^*–/–*^ mice that had been treated with vehicle or 100 μM PTX for 24 h. **c** Amplitudes of mIPSCs (left) and a plot of cumulative event probability as a function of mIPSC amplitude (right) in *Sirt3*^*+/+*^ and *Sirt3*^*–/–*^ neurons treated for 24 h with vehicle or PTX (15 neurons/group). **d** mIPSC frequency (left) and a plot of cumulative event probability as a function of mIPSC inter-event interval (right) in *Sirt3*^*+/+*^ and *Sirt3*^*–/–*^ neurons treated for 24 h with vehicle or PTX (15 neurons/group). *P < 0.05 compared to the values for each of the other 3 groups. **e** Whole-cell recordings of miniature postsynaptic currents (mIPSCs) in cultured hippocampal neurons from *Sirt3*^*–/–*^ mice that had been infected with AAV vectors to overexpress either SIRT3 and GFP, or GFP alone; 6 days after infection, cultures were treated with vehicle or 100 μM PTX for 24 h. **f** Amplitudes (left) and frequencies (right) of mIPSCs in *Sirt3*^*–/–*^ neurons in the four different groups (15 neurons/group). *P < 0.05 compared to the values for each of the other 3 groups. **g** Whole-cell recordings of miniature postsynaptic currents (mIPSCs) in cultured hippocampal neurons from *Sirt3*^*+/+*^ mice that had been treated for 24 h with vehicle, 100 μM PTX, 0.3 μM paraquat (PQ) plus PTX, 1.0 μM PQ plus PTX, or 0.3 μM PQ plus vehicle. **h** mIPSC amplitudes (left) and frequencies (right) in *Sirt3*^*+/+*^ neurons in the indicated treatment groups (24 h treatment; 12 neurons/group). *P < 0.05. **i** Results of measurements of mIPSC amplitudes in *Sirt3*^*-/-*^ neurons in the indicated treatment groups (24 h treatment; 12 neurons/group). *P < 0.05. All error bars are the SEM. All statistical comparisons for data in this figure were performed using ANOVA and Newman–Keuls post hoc tests for pairwise comparisons. Source data for all graphs in this figure are provided in supplemental Source Data file

Studies of primary hippocampal neurons in culture have shown that an imposed increase in excitatory synaptic activity up-regulates GABAergic neurotransmission to constrain neuronal network activity, a process called synaptic downscaling in which the relative difference in synaptic strengths induced by Hebbian mechanisms is preserved by scaling synaptic strength up or down proportionally^38,39^. Because IF up-regulated GABAergic neurotransmission in a SIRT3-dependent manner, we designed a series of experiments in cultured hippocampal neurons established from *Sirt3*^*+/+*^ and *Sirt3*^*–/–*^ embryos to determine if and how SIRT3 affects the process of synaptic downscaling. Experiments were performed in neurons that had been maintained in culture for 16 days, a time period during which extensive neuronal networks with abundant glutamatergic and GABAergic synapses form^38^. Exposure of cultured neurons to 100 μM picrotoxin induced a significant increase in SIRT3 protein levels within 12–24 h (Fig. 4a). Patch clamp recordings showed that WT neurons treated for 24 h with picrotoxin exhibit a significant increase in the amplitude, but not the frequency, of mIPSCs (Fig. 4b–d). In contrast, picrotoxin had no effect on the amplitude or frequency of mIPSCs in *Sirt3*^*–/–*^ neurons. We next infected neurons from *Sirt3*^*-/-*^ mice with an adeno-associated virus construct engineered to express SIRT3 (AAV-*Gfp-Sirt3*). When the *Sirt3*^*–/–*^ neurons expressing ectopic SIRT3 were incubated for 24 h in the presence of picrotoxin, the amplitudes of mIPSCs were increased significantly compared to *Sirt3*^*–/–*^ neurons infected with control (AAV-*Gfp*) virus (Fig. 4e, f).

Mitochondrial SOD2 is a substrate of SIRT3, and the superoxide dismutase activity of SOD2 is increased when it is deacetylated by SIRT3^24,40^. To determine whether mitochondrial superoxide levels influence synaptic downscaling induced by prolonged exposure to picrotoxin, we exposed neurons to agents known to either increase (paraquat; PQ) or decrease (the SOD mimetic EUK134) mitochondrial superoxide levels^41^. In WT neurons, treatment with 1 μM paraquat abolished the ability of picrotoxin treatment to elevate mIPSC amplitudes (Fig. 4g, h). Interestingly, a lower concentration of paraquat (0.3 μM) alone caused a significant increase of the mIPSC amplitude and did not alter the ability of picrotoxin treatment to increase the mIPSC amplitude (Fig. 4g, h). To further interrogate the potential involvement of SOD2 in synaptic downscaling, we recorded mIPSCs in neurons in cultures established from *Sod2*^*+/–*^ mouse embryos and wild type littermate embryos. Whereas picrotoxin treatment increased mIPSC amplitudes in wild type neurons, it did not increase mIPSC amplitudes in *Sod2*^*+/–*^ neurons (Supplementary Figure 4a, b). Recordings from *Sirt3*^*–/–*^ neurons showed that co-treatment of the neurons with a low concentration of EUK134 (1 μM), but not a higher concentration of EUK134 (10 μM), restored the ability of picrotoxin to enhance the mIPSC amplitude (Fig. 4i). The ability of picrotoxin treatment to increase SIRT3 protein levels was significantly attenuated in neurons treated with 10 μM EUK134 (Fig. 4a), indicating a role for superoxide-related signaling in the regulation of SIRT3 expression. Collectively, these findings suggest that SIRT3 enhances hippocampal GABAergic tone by a mechanism involving mitochondrial superoxide production.

## Discussion

Our data reveal a previously unknown mitochondrial SIRT3-mediated mechanism by which hippocampal neuronal networks adapt to IF by up-regulating GABAergic synaptic activity. We elucidate roles for these mitochondrial and synaptic adaptations in the anxiolytic and cognition-enhancing effects of IF. Moreover, the results of experiments with IF in *App*^*NL-G-F*^ mice suggest a translational potential for IF in mitigating synaptic and cognitive deficits in AD. Maintenance of mice on an IF feeding schedule ameliorated anxiety-like responses to acute food deprivation with preservation of cognitive performance. IF prevented the hyperexcitability of hippocampal and amygdala neurons that occurs in response to acute food deprivation in mice previously fed AL. Our patch-clamp recordings from hippocampal neurons in brain slices and cultured neurons from wild type and SIRT3-deficient mice established that the regulation of neuronal network excitability by IF is mediated by increased inhibitory (GABAergic) synaptic activity and requires the mitochondrial protein deacetylase SIRT3. Mice lacking SIRT3 in hippocampal neurons did not adapt to long term IF as indicated by heightened anxiety and relatively poor memory retention in behavioral tests, and by impaired long-term potentiation at hippocampal synapses.

Evolution selects for individuals whose brains and bodies perform at high levels and are relatively resistant to stress when in a fasted state^42^. But in contrast to the evolutionarily normal situation of intermittent food availability, most individuals in modern human societies have constant access to food and so eat multiple meals throughout the day. We found that mice previously fed AL exhibit anxiety-like behaviors when deprived of food for 24 h, whereas mice adapted to IF exhibit no such anxiety response. Moreover, the mice on IF exhibited a generalized stress resistance as demonstrated by an attenuated neuroendocrine stress response as indicated by reduced elevation of serum corticosterone concentrations. Emerging findings suggest that behavioral adaptations of humans that transition from AL to an IF eating pattern are similar to those of mice. Humans initially experience hunger and mood disturbances on the fasting days, but these initial ‘side effects’ resolve within 2–4 weeks and subjects report a stable positive mood thereafter^3,6^. It will be of considerable interest to evaluate brain neuronal network activity using functional magnetic resonance imaging in human subjects as they adapt to an IF feeding schedule.

It was previously reported that hippocampal neurons of rats or mice adapted to IF are resistant to seizure-induced damage in an epilepsy model^21^. Subsequent studies confirmed that IF constrains neuronal excitability in the hippocampus^43,44^, and can also protect neurons in other brain regions against metabolic and excitotoxic stress in rodent models of stroke^45,46^, Parkinson’s disease^47^ and Huntington’s disease^48^. Mechanisms underlying the neuroprotective effect of IF may include up-regulation of expression of neurotrophic factors and antioxidant defenses, and suppression of inflammation^1,23^. Our findings suggest an additional mechanism in which IF results in up-regulation of GABAergic synaptic activity to constrain neuronal excitability. Moreover, the results of our measurements of hippocampal mIPSCs in brain slices, and behavioral testing of *Sirt3*^*–/–*^ mice, and wild type mice in which SIRT3 was depleted from hippocampal neurons, demonstrate an essential role for SIRT3 in the neuronal network modifying, anxiolytic and cognition sparing effects of IF.

Mitochondrial reactive oxygen species play important signaling roles when generated transiently in low to moderate levels, but can cause synaptic dysfunction and neuronal degeneration when generated in larger amounts over an extended time period^29,49^. Studies of hippocampal synaptic plasticity in brain slice preparations have provided evidence that long-term potentiation of synaptic transmission requires superoxide production^50,51^. We found that when SOD2 expression is reduced (*Sod2*^*+/-*^ mice), the adaptive up-regulation of inhibitory synaptic activity that normally occurs in response to IF was diminished. In addition, we found that levels of SIRT3 increase by approximately 2-fold in hippocampal cells in mice adapted to IF and SIRT3 is required for maintenance of hippocampal synaptic plasticity (LTP) and hippocampus-dependent memory retention in mice adapted to IF. It was previously established that deacetylation of SOD2 by SIRT3 increases the enzymatic activity of SOD2, and SOD2 is deacetylated by SIRT3 in hippocampal neurons^24,52^. Our data therefore suggest a mechanistic links between SIRT3, its stimulation of SOD2 activity, and the regulation of hippocampal neuronal network activity and associated behavioral adaptations by IF.

Studies of human subjects and animal models suggest that neuronal hyperexcitability in AD occurs early in the disease process, prior to the degeneration and death of neurons in the affected neuronal networks^34,36,53,54^. Previous findings suggest a role for reduced GABAergic synaptic activity in the hyperexcitability of vulnerable neuronal networks in AD^55,56^, and treatment with GABA receptor agonists can restore synaptic plasticity and cognitive function in mouse models of AD^57,58^. We found that IF suppresses the development of seizures, enhances hippocampal synaptic plasticity, and ameliorates spatial learning and memory deficits in *App*^*NL-G-F*^ mice. The *App*^*NL-G-F*^ mice provide a useful model for investigating mechanisms by which age-related accumulation of Aβ impairs synaptic plasticity and cognition^25^. However, the *App*^*NL-G-F*^ mice do not exhibit neurofibrillary tangles, presumably because they express mouse and not human Tau. We found that knockdown of SIRT3 caused hyperexcitability of hippocampal neurons in the *App*^*NL-G-F*^ mice, even in the absence of an imposed stress. The latter finding suggests an important role for SIRT3 in protecting neurons against Aβ-induced excitotoxicity. It was previously reported that, when initiated prior to the accumulation of Aβ, IF can forestall the onset of cognitive impairment during aging in the 3xTgAD mouse model of AD^59^. In the present study, IF was initiated in 1 year-old mice an age at which considerable accumulation of Aβ had already occurred and at which cognitive deficits are already evident^37^. Our findings therefore suggest that IF can reverse pre-existing deficits in hippocampal synaptic plasticity and cognition by a mechanism involving SIRT3-mediated suppression of hyperexcitability. While IF suppressed hippocampal neuronal excitability in *App*^*NL-G-F*^ mice, we found only a non-significant trend towards reduced Aβ load in those AD mice. Because previous cell culture studies have shown that Aβ increases the vulnerability of neurons to excitotoxicity^35^, our findings suggest that IF protects neurons in *App*^*NL-G-F*^ mice against an excitatory imbalance caused by the accumulation of Aβ.

It was recently reported that aerobic exercise induces SIRT3 expression in cerebral cortical and hippocampal cells^24^. In light of our findings that the beneficial effects of IF on anxiety and cognition are mediated by SIRT3, it will be of interest to determine whether SIRT3 also mediates the anxiolytic and cognition-enhancing effects of exercise^58,60,61^. Individuals who exercise have a reduced risk of AD^62^, and recent findings suggest that initiation of an exercise programs can improve cognition in elderly subjects at risk for AD and patients with mild cognitive impairment and early AD^63,64^. Studies of animal models suggest that there is considerable overlap in the cellular and molecular mechanisms by which IF and exercise enhance brain function and resistance to stress and disease^1^. It will therefore be of considerable interest to perform clinical trials of IF in human subjects at risk for AD or in the early stages of AD.

## Methods

### Mice

All experiments were initiated in male mice. Original breeding pairs of *Sod2*^*+/–*^ mice were purchased from Jackson Laboratories (Bar Harbor, ME; #002973), and original breeding pairs of SIRT3^+/–^ mice were a generous gift from D. Gius (then at the National Cancer Institute and now at Northwestern University). Both the *Sirt3*^*+/–*^ or *Sod2*^*+/–*^ mice have been backcrossed to C57BL/6 mice for at least 10 generations and so were considered congenic. Colonies were maintained by breeding *Sirt3*^*+/–*^ males with *Sirt3*^*+/–*^ females, and *Sod2*^*+/–*^ males with or *Sod2*^*+/–*^ females. *App*^*NL-G-F*^ mice were from RIKEN institute^25^.

All experiments were performed on littermate mice randomly assigned to control or intervention (intermittent fasting (IF) diet or picrotoxin) groups. Mice were housed 3–5 per cage with constant access to water and on a 12 h light/ 12 h dark cycle. For genotyping of pups or embryos in litters from Sirt3^+/–^ dams, two complementary PCRs were performed on the genomic DNA extracted from tails of pups or embryos. The first reaction used primers (forward: 5′ATCTCGCAGATAGGCTATCAGC3′ and reverse: 5′AACCACGTAACCTTACCCAAGG3′), which flank the insertion site of *Sirt3* and produce a 336 bp PCR fragment for wild type (*Sirt3*^*+/+*^) and heterozygous (*Sirt3*^*+/–*^) mice. The second reaction primer set (forward: 5′CTGTGCTCGACGTTGTCACTG3′; reverse: 5′GATCCCCTCAGAAGAACTCGT3′) targets the Neo marker in the insertion element and produces a 556 bp fragment in *Sirt3*^*+/–*^ and homozygous (*Sirt3*^*–/–*^) mice. For genotyping of pups or embryos in litters from *Sod2*^+/–^ dams, two complementary PCRs were performed on genomic DNA with the first reaction used primers for detecting a *Sod2* exon 1 sequence (5′ACGTTGCCTTCCCAGGAT3′ and 5′GTTTACACGACCGCTGCTCT3′) and the second reaction used primers for detecting a mutated sequence (5′TGT TCT CCT CTT CCT CAT CTC C3′ and 5′ACC CTT TCC AAA TCC TCA GC3′). The first reaction generates a 193 bp product for the wild type allele and the second reaction generates a 240 bp product for the mutant allele. All animal procedures were approved by the Animal Care and Use Committee of the National Institute on Aging Intramural Research Program and complied with NIH guidelines.

### IF or drug administration and behavioral tests

Mice were fed a standard NIH-07 diet (Harlan-Teklad, Indianapolis, IN). All mice were maintained with ad libitum (AL) diet before any specific experiment. For IF, food was removed from the hopper at 1700 hours to initiate the fasting day, and then added to the hopper at 1700 hours the following day to begin the feeding day. All behavioral tests were performed between 1800 and 2200 hours. Picrotoxin and diazepam were prepared as concentrated stocks in dimethylsulfoxide, saline and 1% Tween 20, respectively. Picrotoxin was administered by intraperitoneal injection (1.5 or 3.0 mg/kg picrotoxin) and control mice were injected with an equivalent volume of vehicle (0.01% dimethylsulfoxide in saline). Mice received an injection of saline (0.2 ml) on each of the two days preceding drug administration to reduce any potential impact of handling stress on behavioral end-points. For the analysis of the effects of picrotoxin on c-Fos expression analyses, mice were injected with picrotoxin or vehicle 2 h prior to sacrifice. For long-term intermittent picrotoxin treatment, mice were injected twice with picrotoxin (1.5 mg/kg) at 11 and 5 pm every other day. For behavioral testing, mice were injected twice with picrotoxin (1.5 mg/kg) at 7 and 1 h prior to testing. Diazepam (2.5 mg/kg) was administered intraperitoneally 1 h prior to tests. For specific knockdown of SIRT3 in hippocampal neuron, adeno-associated virus serotype 9 expressing SIRT3 siRNA or scrambled siRNA (ABM, BC, Canada) was injected into both the dorsal hippocampus (2 mm posterior, 1.25 mm lateral, 1.25 mm ventral from the bregma) and ventral hippocampus (2.92 mm posterior, 3 mm lateral, 3.8 mm ventral from the bregma) hippocampus of 2-month old WT male mice. All behavior tests were performed 1 month after the injections. In five 18 months old *App*^*NL-G-F*^ mice, intrahippocampal CA1 (2 mm posterior, 1.25 mm lateral, 1.25 mm ventral from the bregma) injection of 0.5 µL lentivirus express SIRT3 siRNA or control siRNA (Santa Curz, CA) was performed separately at one side or other side of hippocampi in the same mouse, by using glass pipette (Sutter Instrument, CA, USA). For knockdown of SIRT3 in *App*^*NL-G-F*^ IF mice which have received 8 months IF, 0.5 µL lentivirus express SIRT3 siRNA or control siRNA (Santa Curz, CA, USA) was injected in bilateral hippocampal CA1 (2 mm posterior, 1.25 mm lateral, 1.25 mm ventral from the bregma) in the mice. One month later, acute brain slices were cut for hippocampal LTP analysis or Golgi staining.

For testing of the IF groups, the term IF NFD denotes testing performed on a feeding day and IF FD means testing performed on a food deprivation day. For elevated plus maze testing, the mouse was placed in the center of an elevated plus maze (EPM) made of white Plexiglas supported by a 60 cm high stand; the dimensions of each arm were 30 cm × 5 cm, with the closed arms having a 16 cm high wall, and a center area of 5 cm × 5 cm. A mouse was placed in the center area and allowed to explore freely for 5 min; the time spent and distance traveled in the open and closed arms were determined using video tracking software (ANY-maze, Stoelting, Wood Dale, IL, USA).

For open field testing, mice were placed individually in an open-field (OF) arena (27.3 × 27.3 cm, height 20.3 cm) housed within a sound-attenuating cubicle (Med Associates, St Albans, VT, USA) and permitted to move freely. Trials lasted for 15 min. Animal motion and cumulative path length were automatically tracked via three 16-beam infrared array and recorded by Activity Monitor software (Version 4.0, Med Associates).

A 2-day water maze testing protocol was used to evaluate spatial learning and memory. The pool was 160 cm in diameter and filled with water made opaque using nontoxic white paint; a 153 cm^2^ platform was located midway between the pool center and wall. On day 1 the platform was positioned 1 cm above the water level and the mouse was placed in the pool four times (4 trials) with a 1-h inter-trial interval. On day two the platform was positioned 1 cm below the water level and the mouse was placed in the pool three times (3 trials) with a 1-h inter-trial interval. For each trial the mouse was placed in the pool facing the wall, with start locations varying pseudorandomly. Each trial lasted a maximum of 180 s. Mice were videotaped and escape latencies determined using ANY-maze software (Wood Dale, IL, USA). For the IF groups, testing is on feeding day.

For the Y-maze test, mice were caged in a group of three to five individuals before transferring to a behavioral laboratory. The Y-maze apparatus (Med Associates), consisted of three compartments (3 cm (W) bottom and 10 cm (W) top, 40 cm (L) and 12 cm (H)) radiating out from the center platform (3 × 3 × 3 cm triangle), and positioned 60 cm above the floor. In this test, each mouse was placed in the center of the maze facing toward one of the arms and then allowed to explore freely for 5 min. The light intensity of the platform was kept at 80 lx. We recorded and analyzed the activity and spontaneous behavioral alternations of the mice using ANY-maze software (Wood Dale, IL). For the IF groups, testing was performed on feeding day.

### Evaluation of seizure-like behavior

In preliminary observations of mice in our colonies, we observed the sudden death of several App^NL-G-F^ mice that exhibited seizure-like behaviors immediately prior to their death; those mice were approximately 80 weeks old. We therefore decided to observe on a daily basis all wild type and App^NL-G-F^ mice in the present study. Mice were scored as having seizures if they exhibited forelimb clonus preceded by head nodding. Mice exhibiting seizure-like behaviors that did not die contemporaneously with the seizures were euthanized when they subsequently exhibited rapid weight loss. Upon death, brains were removed and processed for immunoblot and immunohistochemical analyses. To confirm the seizures, c-fos immunoreactivity staining of brain tissue sections was performed.

### Primary hippocampal neuronal cultures and treatments

Hippocampal cultures were prepared from embryonic day (E) 16 hippocampal tissue. Briefly, pregnant mice were euthanized, embryo brains were removed, and the hippocampus was dissected in sterile Ca^2+^- and Mg^2+^-free Hank’s Balanced Saline Solution (HBSS). The tail of each embryo was collected for genomic DNA extraction and genotyping. The hippocampal tissue from each embryo was incubated in 0.05% trypsin-EDTA in HBSS for 15 min at 37 °C, and then transferred to MEM + (Minimal Essential Medium supplemented with 10% fetal Clone III bovine serum (HyClone, Logan, UT, USA)) and were dissociated by titration using a fire-polished Pasteur pipet. The dissociated cells were seeded into polyethyleneimine-coated plastic culture dishes or glass coverslips at a density of 40,000 cells/cm^2^. After a 4 h incubation in MEM + to allow for cell attachment, the medium was replaced with Neurobasal medium containing B27 supplements, Glutamax supplement and antibiotic-antimycotic (all from ThermoFisher/GIBCO, Gaithersburg, MD), and 1 mM HEPES (Sigma-Aldrich, St. Louis, MO). For culture maintenance, half of the medium was replaced with fresh the Neurobasal medium every 5 days, and osmolality was maintained at 290 mOsm/kg. All treatments in culture were initiated beginning on culture day 16, except adeno-associated virus (AAV) at culture day 12. The treatments included: picrotoxin, paraquat and dimethylsulfoxide (all from Sigma-Aldrich, St. Louis, MO), and EUK-134 (Cayman Chemical Co., Ann Arbor, MI). Methods for construction and packaging of adeno-associated virus Sirt3 expression vector have been described (*29*). Treatments were administered by direct dilution into the culture medium, and an equivalent volume of vehicle was added to control cultures.

### Electrophysiology

For hippocampal neuronal cultures, whole-cell patch clamp recordings were made from neurons under continuous perfusion of a medium containing (in mM) 119 NaCl, 2.5 KCl, 2 CaCl_2_, 2 MgCl_2_, 26 NaHCO_3_, 1 NaH_2_PO_4_, 11 glucose (osmolarity of 290 mOsm). The medium was gassed with 5% CO_2_/95% O_2_ to maintain oxygenation and a pH of 7.4. To record miniature GABAergic currents (mIPSCs), the patch-clamp electrodes (3–4MΩ) were filled with an internal solution containing (in mM) 115 Cs-methanesulfonate, 20 CsCl, 10 Na_2_-phosphocreatine, 10 HEPES, 0.6 EGTA, 2.5 MgCl_2_, 2 MgATP, 0.3 Na_2_GTP and 0.1 Alexa Fluor 594; pyramidal like neurons were clamped at −70 mV and perfused with medium containing 1 µM TTX, 20 µM NBQX, 50 µM AP5 and 5 µM strychnine. The pyramidal neuron phenotype was confirmed by evaluation of morphology acquired form imaging after recording. Data were collected using a MultiClamp 700B amplifier (Molecular Devices, Sunnyvale CA). Signals were filtered at 2 kHz and digitized at 10 kHz with a Digidata 1550 Data Acquisition System, and analyzed using pCLAMP 10 software (Molecular Devices) and Mini Analysis software (Synaptosoft, Decatur GA). All the detected events (mIPSCs) were re-examined and accepted or rejected on the basis of visual examination. Cells were recorded from for ~5 min to obtain at least 100 events per cell. Recording and data analyses were performed by an investigator (Y. L.) blinded as to the treatment history of the cultures.

For acute hippocampal slices, transverse acute hippocampal slices (400 μm) were cut in chilled (2–4℃) artificial cerebrospinal fluid (ACSF) containing (mM): 92 N-methyl-D-glucamine, 2.5 KCl, 1.25 NaH_2_PO_4_, 30 NaHCO_3_, 20 HEPES, 25 glucose, 2 thiourea, 5 Na-ascorbate, 3 Na-pyruvate, 0.5 CaCl_2_ and 10 MgSO_4_ (pH 7.3–7.4). The slices were then transferred to an incubator for recovery for ~10 min at 32–34 °C. After the initial recovery period, the slices were transferred to a new holding incubator (room temperature) with HEPES ACSF containing (mM): 92 NaCl, 2.5 KCl, 1.25 NaH_2_PO_4_, 30 NaHCO_3_, 20 HEPES, 25 glucose, 2 thiourea, 5 Na-ascorbate, 3 Na-pyruvate, 2 CaCl_2_ and 2 MgSO_4_, for 1 h. The slices were then transferred to a recording chamber perfused with standard ACSF containing (mM): 119 NaCl, 2.5 KCl, 1.25 NaH_2_PO_4_, 24 NaHCO_3_, 12.5 glucose, 2 CaCl_2_ and 2 MgSO_4_. All ACSF solutions were saturated with 95% O_2_/5% CO_2_ prior to use to ensure a stable pH and adequate oxygenation.

The methods used record miniature GABAergic currents (mIPSCs) from hippocampal CA1 pyramidal neurons in hippocampal slices were identical to those used to record IPSCs from dissociated hippocampal neurons in culture. Field potential recordings were performed at 30–32 °C in slices in a perfusion chamber. For Only slices in which field recordings exhibited a steep input-output curve were included. Field excitatory postsynaptic potentials (fEPSPs) were recorded in CA1 stratum radiatum with stimuli (30μs duration every 20 s) delivered with a bipolar tungsten electrode to activate Schaffer collateral/commissural afferents. LTP was induced with a train of tetanic stimulation (100 Hz for 1 s). Plots were normalized to the initial slope of the EPSPs with each data point representing the averaged values during a 1-minute period (three consecutive sweeps with an interval of 20 s). Recording and data analyses were performed by an investigator (Y. L.) blinded as to the treatment history of the cultures. For fEPSCs recording in hippocampal slice from old *App*^*NL-G-F*^ and WT mice, recording and data analyses of were performed by an investigator (Y. W.) blinded as to the groups.

*Immunoblot analysis*: Cultured cells or tissues were solubilized in SDS sample buffer, and the protein concentration in each sample was determined using a BioRad protein assay kit (BioRad, Hercules CA) with bovine serum albumin as the standard. Proteins (30 µg of protein per lane) were separated electrophoretically was performed using 4–10% SDS gradient polyacrylamide gel followed by a protein transfer and blotting procedure. The primary antibodies included those that selectively recognize: SIRT3 (1:1000; Cell Signaling Technologies, Danvers MA) and actin (1:2000; Sigma-Aldrich).

*Immunohistochemistry, and image acquisition and analysis*: Mice were anesthetized and perfused transcardially with PBS, followed by 4% paraformaldehyde in PBS (pH 7.4). Brains were post-fixed overnight and then incubated for 1–3 days in a solution of 30% sucrose in PBS at 4 °C, and then stored at −80 °C. Cryostat sections were cut in the transverse plane at a thickness of 40 µm. Free-floating sections were immunostained with a c-Fos antibody as follows. Sections were permeabilized by incubation in 0.3% Triton X-100 in PBS (1 h), incubated for 60 min in blocking solution (0.3% Triton X-100, 1% BSA, 10% normal goat serum in PBS) and then incubated for 24 h at 4 °C with c-Fos antibody (Cell Signaling, Cat no. 2250, MA) diluted 1:400 in a solution of PBS containing 0.3% Triton X-100 and 1% BSA. Slices were washed with PBS and incubated with FITC goat anti-rabbit IgG secondary antibody (1:500) for 1 h at room temperature. Images of c-Fos immunoreactivity were acquired and analyzed using a DeltaVision system (GE Healthcare Lifesciences, Pittsburgh PA) on an Olympus IX71 inverted microscope and Image J software (NIH, Bethesda MD). Counts of c-Fos positive neurons were made in the basolateral amygdala (BLA), the central amygdala nucleus (CE), hippocampal CA1 and CA3 pyramidal neurons, and dentate granule neurons bilaterally in sections from 5 mice per group (3 sections analyzed per mouse). The sections used for the analyses were from the following rostro-caudal levels (amygdala, 1.46–1.70 mm caudal from bregma; hippocampus, 1.94–2.18 mm caudal from bregma). All other images were acquired using the confocal with an Olympus confocal laser scanning microscope. All cell counts were performed by an investigator blinded as to the genotype and treatment history of the mice.

For detection the amyloid β-peptide (Aβ) plaques, a monoclonal antibody raised against Aβ (Cell Signaling, Cat no. 14974, MA) was used. Slides were incubated with boiling citric acid, quenched in 0.3% peroxide solution and blocked with phosphate-buffered saline with bovine serum albumin and Tween. Primary antibody (1:200) were applied overnight followed by treatment with a biotinylated goat anti-rabbit immunoglobulin G. Sections were developed using 3,3-diaminobenzidine (Vector Labs; Burlingame, CA) dehydrated in an ethanol series, and cover-slipped using Permount (Fisher Chemical, Fairlawn, NJ). A negative control with no primary antibody was always included for verification of specificity of immunohistochemical techniques. Aβ staining in the hippocampus was analyzed using quantitative densitometry. Hippocampi were photographed at 10× magnification using a digital camera and stereomicroscope system and AxioVision software, version 4.8.2 (Zeiss, Thornwood, NY). Images were cropped to include the region of interest. The percentage of the area of each microscope field occupied Aβ immunoreactivity was then quantified using Image J software (NIH, Bethesda MD).

### Serum chemistry

3-hydroxybutyrate concentrations were quantified in serum using a Roche Cobas Fara II analyzer (Roche Diagnostic Systems; Montclair, NJ). Corticosterone concentrations were quantified in serum using Corticosterone EIA Kit (Arbor Assays Inc, MI).

### Golgi staining and quantification of dendrite spine density

Golgi staining was performed using a rapid Golgi staining kit (FD NeuroTechnologies, MD). Briefly, 200 µm acute hippocampal slices were prepared as described above. Slices were quickly rinsed with distilled water and prepared for Golgi staining by following the manufacturer’s manual. Images of dendritic spines were acquired using a 100 × objective. For each hippocampal CA1 pyramidal neuron, the spines in the principal apical dendrite were counted in 50–100μm segments that were at least 50μm away from the cell body of the neuron, and a 30μm segment of secondary apical dendrite. Spines were counted only if they had both a head and visible neck. Analyses were performed in a blinded manner. A subset of neurons was counted by two different investigators to ensure consistency of counting. No significant differences were found when the same segment was counted by different investigators.

### Statistical analyses

Statistical analyses were performed by two-way ANOVA with repeated measures, one-way ANOVA or Student’s *t* test using GraphPad Prism (GraphPad Software, CA, USA). Student Newman–Keuls post hoc tests were applied to detect statistical differences between groups. Data are expressed as mean ± SEM, and a *P* value < 0.05 was considered statistically significant.

## Supplementary information

Supplementary Information

Source Data

## Data Availability

Source data for all graphs in the figures of this manuscript are provided in supplemental Source Data file. Any other data are available from the corresponding authors upon reasonable request.
