# Supplementary material for: SIRT3 mediates hippocampal synaptic adaptations to intermittent fasting and ameliorates deficits in APP mutant mice
Source: Nat Commun. 2019 Apr 23;10:1886. doi: 10.1038/s41467-019-09897-1 (PMC6478744; doi:10.1038/s41467-019-09897-1)
Supplement: Supplementary file 1 — Supplementary Information [file 41467_2019_9897_MOESM1_ESM.pdf]

Supplementary Information

Liu et al.

SIRT3 Mediates Hippocampal Synaptic Adaptations to Intermittent Fasting and Ameliorates Deficits in APP Mutant Mice

Supplementary Figures

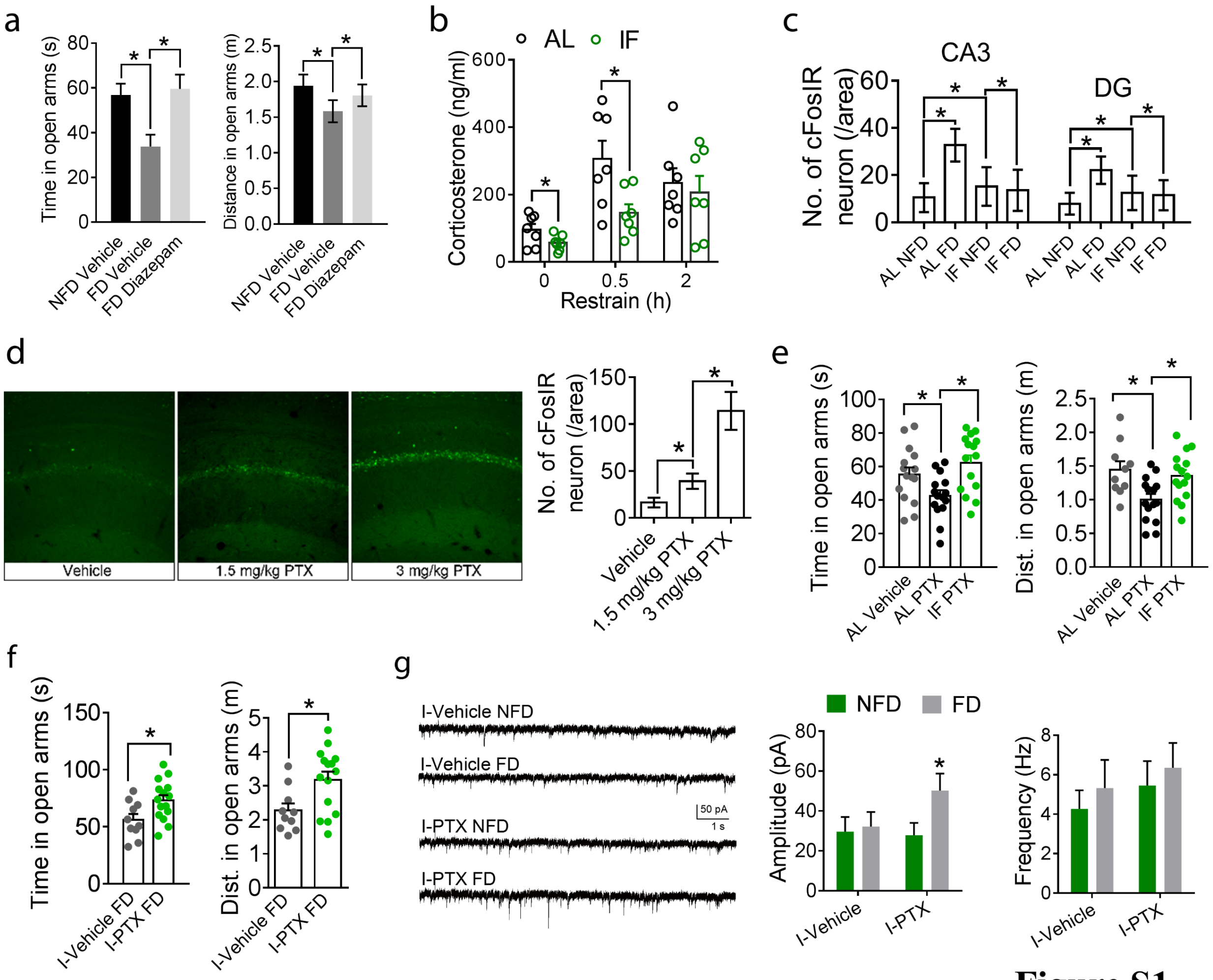

Figure S1

Supplementary Figure 1. Behavioral adaptation to IF involves enhancement of GABAergic tone and reduced hippocampal neuronal network excitability. (**a**) Diazepam (2.5 mg/kg, i.p., 1 h before behavior test) ameliorates anxiety-like behavior induced by food deprivation. WT mice were treated with vehicle

or diazepam (2.5 mg/kg) and then either deprived of food for 24 hours (FD) or not deprived of food (NFD). Mice were then tested in the elevated plus maze (10 mice/group). *P<0.05. (**b**) Corticosterone concentrations in the serum of mice that had been maintained on AL or IF diets for 1 month. Serum samples were taken prior to and 0.5 and 2 hours after the onset of restraint stress. *P<0.001. (**c**) Results of quantification of c-Fos immunoreactive neurons in regions CA3 and dentate gyrus (DG) of mice that had been maintained for 1 month on either AL or IF diets, and then either deprived of food for 24 hours (FD) or not deprived of food (NFD). *P<0.05. (**d**) Examples of images of c-Fos immunoreactivity in hippocampal CA1 neurons of mice that had been administered vehicle, or either 1.5 or 3.0 mg/kg picrotoxin (PTX) 2 hours prior to sacrifice. The graph shows the results of counts of cFos immunoreactive (cFosIR) neurons in mice in the three indicated groups (5 mice/group). (**e and f**) Results of measurements of time spent (left) and distance traveled (right) in the open arms of the elevated plus maze for mice in the indicated groups. The numbers of mice per group for the data in panel E were: AL vehicle, 10; AL PTX, 15; IF PTX, 15. The numbers of mice per group for the data in panel F were: I-vehicle (1 month intermittent vehicle injection) FD, 10; I-PTX (1 month intermittent 1.5 mg/kg picrotoxin injection) FD, 15. *P<0.05, **P<0.01. (**g**) Representative whole-cell recordings of mIPSCs in CA1 neurons in hippocampal slices from mice in the four indicated groups (I-Vehicle NFD, I-Vehicle FD, I-PTX NFD, I-PTX FD). Results of measurements of mIPSC amplitudes and mIPSC frequency in response to FD (data are from 13 neurons from 5 mice/group). *P<0.05 compared to the values for each of the other three groups. All error bars are the SEM. Student's t test was used for analyses of data in panel f. ANOVA and Newman-Keuls post-hoc tests were used for analyses of data in panels a-e, g. Source data for all graphs in this figure are provided in supplemental Source Data file.

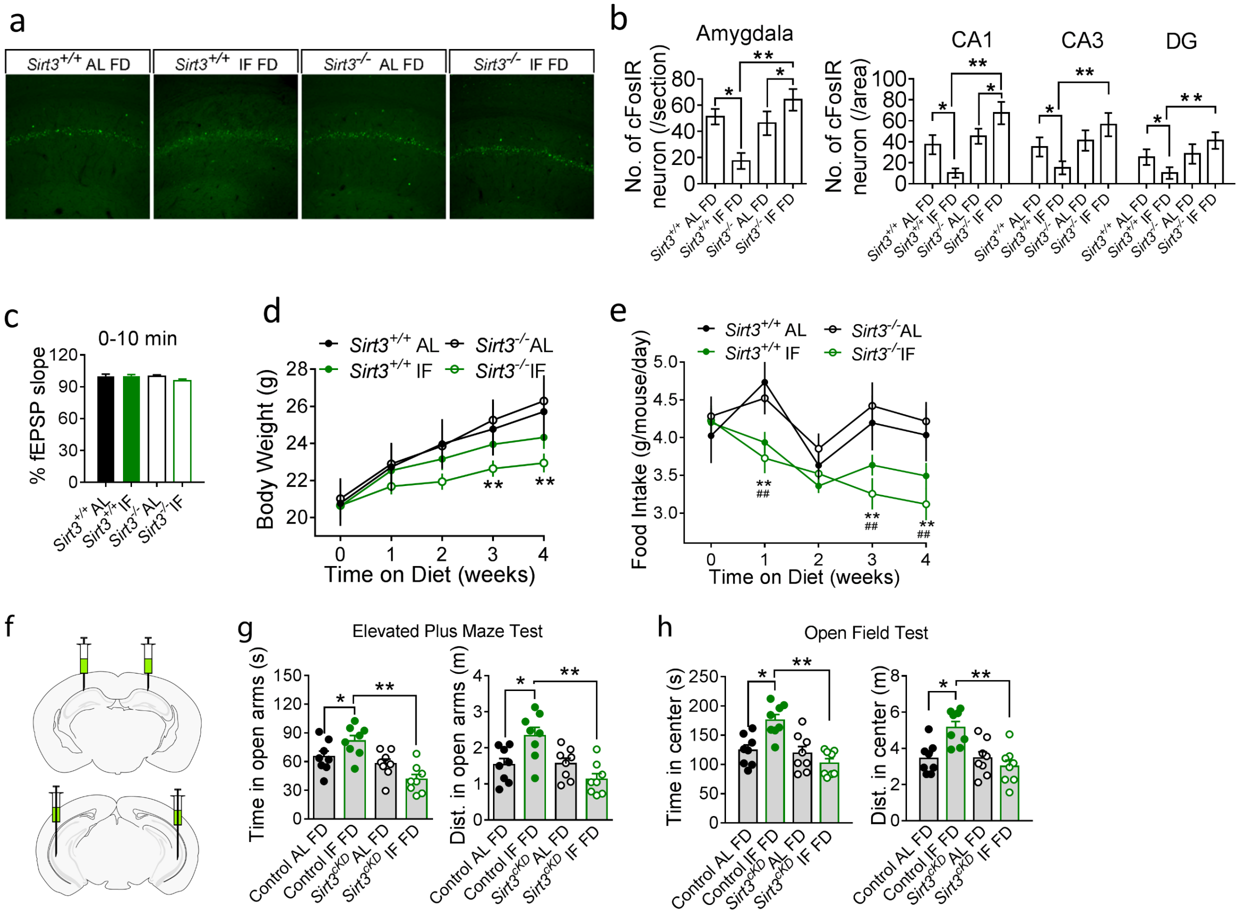

Figure S2

Supplementary Figure 2. SIRT3 is required for the adaptive behavioral and neuronal network responses to IF. (**a**) Examples of images of c-Fos immunoreactivity in hippocampal CA1 neurons of *Sirt3*$^{+/+}$ and *Sirt3*$^{-/-}$ mice that had been maintained for 1 month on either AL or IF diets and then subjected to FD for 24 hours. (**b**) Results of counts of c-Fos immunoreactive (cFosIR) neurons in the amygdala, and CA1, CA3 and dentate granule neurons of the hippocampal CA1, in mice in the four indicated groups (5 mice/group). **P*<0.05, ***P*<0.01. (**c**) The results of measurements of short-term potentiation during the first 10 minutes after frequency stimulation (10 slices from 5 mice/group). **(d)** Body weight of mice in the indicated groups during a 1month period after diet initiation. ***P*<0.01 compared to other three groups (8 mice/group). **(e)** Food intakes of mice in the indicated groups during a 1month period after diet initiation. ***P*<0.01 *Sirt3*$^{+/+}$ IF compared to *Sirt3*$^{+/+}$ AL, $^{\#\#}$*P*<0.01 *Sirt3*$^{-/-}$ IF compared to *Sirt3*$^{-/-}$ AL. (f) Schematic diagram showing sites within the hippocampus where adeno-associated virus for SIRT3 conditional knockdown (*Sirt3*^{cKD}) was injected. (g) The results of measurements of time spent (left) and distance traveled (right) in the open arms of the elevated plus maze for mice in the indicated groups. (h) The results of measurements of time spent (left) and distance traveled (right) in the center of the open field for mice in the indicated groups. 8 mice/group, **P*<0.05. All error bars are the SEM. All statistical comparisons for data in this figure were performed using ANOVA and Newman-Keuls post hoc tests for pairwise comparisons. Source data for all graphs in this figure are provided in supplemental Source Data file.

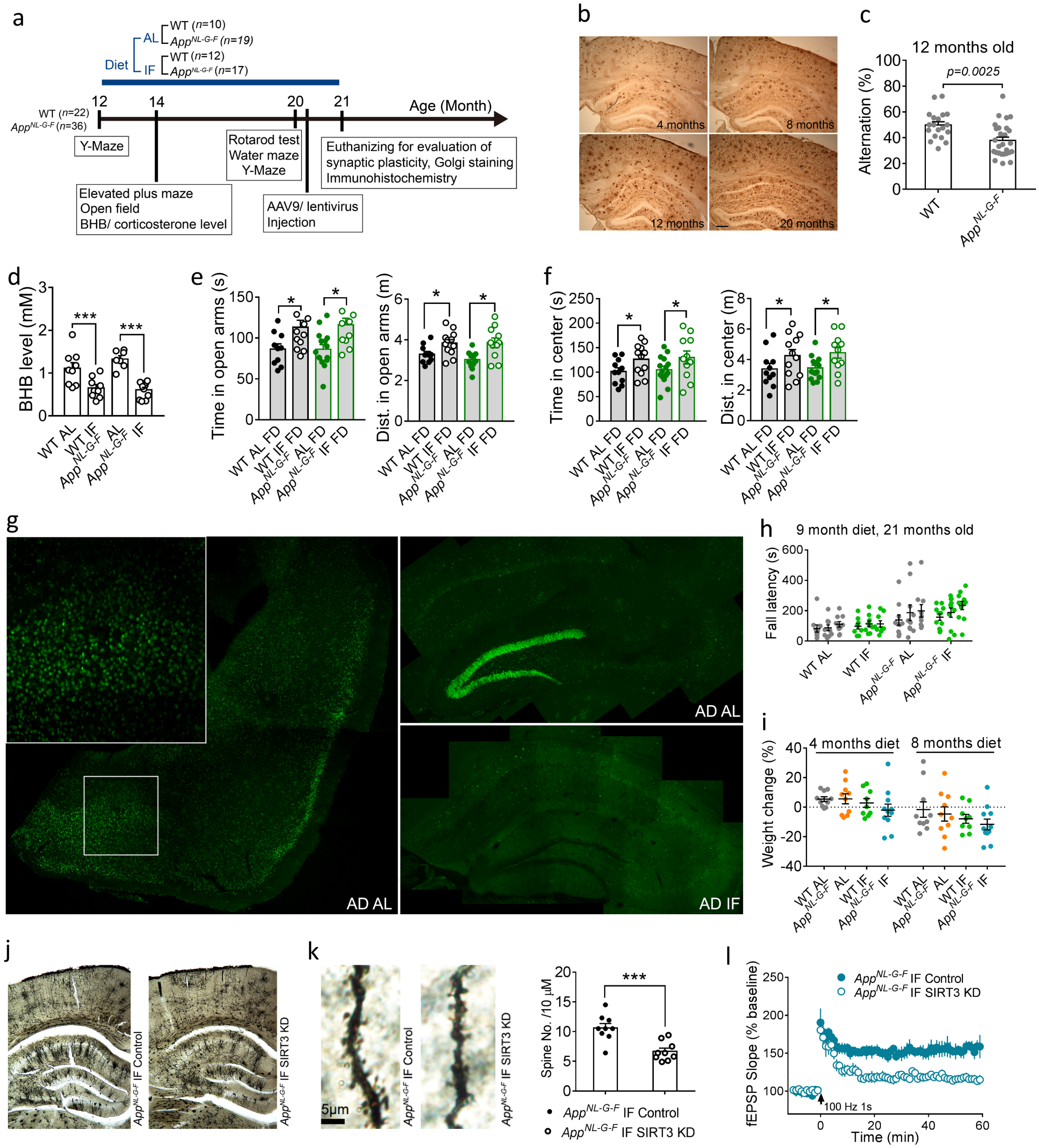

Figure S3

Supplementary Figure 3. IF prevents seizures and ameliorates deficits in cognition and synaptic plasticity impairment in the *App*$^{NL\text{-}G\text{-}F}$ knock-in mouse model**.** (**a**) Schematic diagram showing the experimental timeline, including numbers of mice used for each analysis. (**b**) Examples of images of Aβ immunoreactivity in hippocampus of *App*$^{NL\text{-}G\text{-}F}$ mice at 4, 8, 12, 20 months of age (scale bar, 0.2 mm). (**c**) Results of Y-maze testing in 1 year old WT and *App*$^{NL\text{-}G\text{-}F}$ mice. (**d**) BHB levels in serum after overnight food deprivation. ***P<0.001. (**e and f**) The results of measurements of time spent (left) and distance traveled (right) in the center area of the open field for mice in the indicated groups (e) and time spent (left) and distance traveled (right) in the open arms of the elevated plus maze for mice in the

indicated groups (f). WT AL, 10 mice; WT IF, 12 mice; *App*$^{NL\text{-}G\text{-}F}$ AL, 15 mice; *App*$^{NL\text{-}G\text{-}F}$ IF, 11 mice. FD, food deprivation. *P<0.05. (**g**) Examples of images of c-fos immunoreactivity in cortex (left) and hippocampus (right) of *App*$^{NL\text{-}G\text{-}F}$ mice under ad libitum diet (*App*$^{NL\text{-}G\text{-}F}$ AL) with observed seizures, and *App*$^{NL\text{-}G\text{-}F}$ mice under IF diet (*App*$^{NL\text{-}G\text{-}F}$ IF). (**h**) Body weights of mice a 4 8 months after diet initiation. (**i**) Fall latencies in Rotarod testing of mice in the indicated groups. (**j**) Representative Golgi staining images of hippocampus and dendritic spine in *App*$^{NL\text{-}G\text{-}F}$ IF mice with SIRT3 knockdown and control. (**k**) Representative dendritic spines of Golgi stained CA1 neurons (left) and spine densities (right) in the indicated groups. 3 mice/group. ***P<0.001. **(l)** The results of LTP analysis in indicated groups. 6 slices from 3 mice each group. Group difference, F (1, 10) = 8.561, P=0.0151. All error bars are the SEM. Student's t test was used for analyses of data in panels c and k. ANOVA and Newman-Keuls post-hoc tests were used for analyses of data in panels d-i and 1. Source data for all graphs in this figure are provided in supplemental Source Data file.

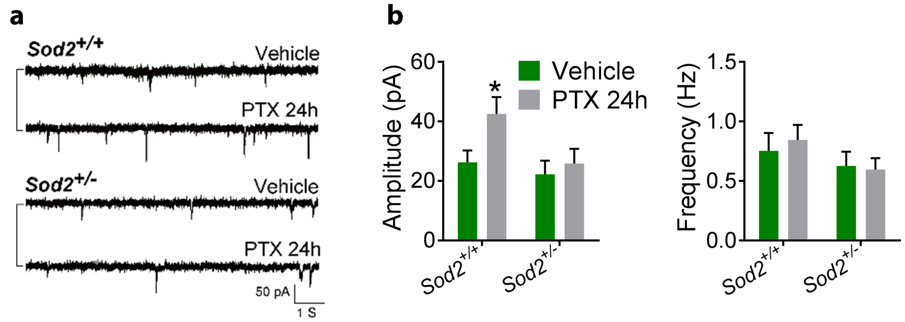

Figure S4

Supplementary Figure 4. GABAergic synaptic scaling is impaired in *Sod2*$^{+/-}$ neurons. (**a**) Representative whole-cell recordings of miniature postsynaptic currents (mIPSCs) in cultured hippocampal neurons from *Sod2*$^{+/+}$ mice and *Sod2*$^{+/-}$ mice that had been treated for 24 hours with vehicle or 100 μM PTX are shown at the left. (**b**) Results of measurements of mIPSC amplitudes and frequencies in neurons in the indicated groups (15 neurons/group). *P<0.05 compared to each of the three other groups. All error bars are the SEM. All statistical comparisons for data in this figure were performed using ANOVA and Newman-Keuls post hoc tests for pairwise comparisons. Source data for all graphs in this figure are provided in supplemental Source Data file.
